# Prevalence of heavy menstrual bleeding and associations with physical health and wellbeing in low-income and middle-income countries: a multinational cross-sectional study

**DOI:** 10.1016/S2214-109X(23)00416-3

**Published:** 2023-10-03

**Authors:** Sheela S Sinharoy, Lyzberthe Chery, Madeleine Patrick, Amelia Conrad, Anupama Ramaswamy, Aparna Stephen, Jenala Chipungu, Y Malini Reddy, Rinchen Doma, Sant-Rayn Pasricha, Tanvir Ahmed, Chibwe Beatrice Chiwala, Niladri Chakraborti, Bethany A Caruso

**Affiliations:** a Hubert Department of Global Health, Rollins School of Public Health, Emory University, Atlanta, GA, USA; b Gangarosa Department of Environmental Health, Rollins School of Public Health, Emory University, Atlanta, GA, USA; c Department of Behavioral, Social, and Health Education Sciences, Rollins School of Public Health, Emory University, Atlanta, GA, USA; d Athena Infonomics, Chennai, India; e Centre for Infectious Disease Research in Zambia, Lusaka, Zambia; f Population Health and Immunity Division, The Walter and Eliza Hall Institute, Melbourne, VIC, Australia; g Department of Civil Engineering and International Training Network, Bangladesh University of Engineering and Technology, Dhaka, Bangladesh; h Lusaka Water Supply and Sanitation Company, Lusaka, Zambia; i Indian Institute for Human Settlements, Chennai, India

## Abstract

**Background:**

Data on the prevalence of heavy menstrual bleeding in low-income and middle-income countries (LMICs) are scarce. We aimed to assess the validity of a scale to measure heavy menstrual bleeding and calculate its prevalence in southern Asia and sub-Saharan Africa, and to examine associations between heavy menstrual bleeding and health outcomes.

**Methods:**

Between Aug 2, 2021 and June 14, 2022, we surveyed 6626 women across ten cities (Meherpur and Saidpur, Bangladesh; Warangal, Narsapur, and Tiruchirappalli, India; Kathmandu, Nepal; Dakar, Senegal; Nairobi, Kenya; Kampala, Uganda; and Lusaka, Zambia), including questions on demographics, health, and the SAMANTA scale, a six-item measure of heavy menstrual bleeding. We conducted confirmatory factor analysis to assess construct validity of the SAMANTA scale, calculated the prevalence of heavy menstrual bleeding, and used regression analyses to examine associations of heavy menstrual bleeding with health outcomes.

**Findings:**

4828 women were included in the final analytic sample. Factor analysis indicated a one-factor model representing heavy menstrual bleeding. In the pooled analytic sample, 2344 (48·6%) of 4828 women were classified as experiencing heavy menstrual bleeding, and the prevalence was lowest in Dakar (126 [38·3%] of 329 women) and Kampala (158 [38·4%] of 411 women) and highest in Kathmandu (326 [77·6%] of 420 women). Experiencing heavy menstrual bleeding was significantly associated with feeling tired or short of breath during the menstrual period (risk ratio 4·12 (95% CI 3·45 to 4·94) and reporting worse self-rated physical health (adjusted odds ratio 1·27, 95% CI 1·08 to 1·51), but was not associated with subjective wellbeing (β –3·34, 95% CI –7·04 to 0·37).

**Interpretation:**

Heavy menstrual bleeding is highly prevalent and adversely impacts quality of life in women across LMIC settings. Further attention is urgently needed to understand determinants and identify and implement solutions to this problem.

**Funding:**

Bill & Melinda Gates Foundation, United States Agency for International Development, National Institutes of Health.

## Introduction

Heavy menstrual bleeding is an understudied global health challenge with potentially far reaching consequences. The clinical definition of heavy menstrual bleeding is “excessive menstrual blood loss which interferes with a woman's physical, emotional, social, or material quality of life”.^1^, ^2^ Heavy menstrual bleeding can cause iron-deficiency anaemia, which is among the leading causes of years lived with disability in low-income and middle-income countries (LMICs).^3^ Heavy menstrual bleeding is also associated with a range of adverse functional outcomes, including lower productivity and income earning, reduced ability to perform daily activities, and limitations on social life and relationships.^4^, ^5^, ^6^, ^7^

Heavy menstrual bleeding has been assessed using a variety of techniques, including laboratory-based approaches to calculate the volume of menstrual blood loss; visual methods, such as the pictorial blood loss assessment chart; and survey-based methods.^8^, ^9^, ^10^ However, laboratory-based and pictorial methods have little utility outside of controlled clinical settings.^10^ Survey-based methods have broader utility but often include references to pads or tampons, which might not be widely used in many settings globally.^11^, ^12^ Survey instruments can also be overly time consuming or complex, and most have not been rigorously evaluated for validity or have been validated only in specific populations in high-income countries.^8^

Due to the scarcity of practical and user-friendly assessment tools, global prevalence data on heavy menstrual bleeding is limited in scope and quality. For example, an internet-based survey conducted among women in five European countries found that 27·2% of respondents had experienced two or more predefined heavy menstrual bleeding symptoms in the preceding year, but the survey did not include a validated measure of heavy menstrual bleeding.^13^ In a 2004 systematic review, the estimated prevalence of heavy menstrual bleeding in LMICs ranged between 4% and 27%.^14^ However, the authors noted that data were scant and highlighted a need for more precise and reliable questions in future surveys.^14^ Studies in LMICs have found that the prevalence of heavy menstrual bleeding ranged from 4% among women in The Gambia to 45·7% among adolescent girls in rural Tamil Nadu, India.^15^, ^16^, ^17^, ^18^ However, these studies used researcher-created survey instruments and the validity of the instruments was not reported.

**Research in context**

**Evidence before this study**

Heavy menstrual bleeding, by definition, impairs quality of life; however, little is known about the extent of this impairment or the populations affected. We searched PubMed from database inception to Jan 30, 2023, for research articles using the search terms (“heavy menstrual bleeding” OR “menorrhagia” OR “abnormal uterine bleeding” OR “menstrual disorders”) AND (“low and middle income countries” OR “developing country” OR “Asia” OR “Africa”), without language restrictions. Few studies had assessed the prevalence of heavy menstrual bleeding in low-income and middle-income countries (LMICs), and studies that did had used disparate measures that had not been evaluated for validity, and existing data were determined to be of low quality.

**Added value of this study**

This study is the first to collect data on heavy menstrual bleeding in a standardised way across multiple LMIC populations, using a measure that was previously validated and for which we have generated additional evidence of validity in our study populations. Our data suggest that a substantial proportion of people who menstruate experience impairments in quality of life due to their menstruation, making heavy menstrual bleeding a serious and pressing health problem that requires attention across the study settings. This study fills an important gap, providing rigorous evidence and justification for the development of new programmes, policy, and research agendas focused on understanding and addressing the problem of heavy menstrual bleeding.

**Implications of all the available evidence**

Heavy menstrual bleeding is a common and under-studied problem affecting and impairing the quality of life of a substantial proportion of people who menstruate. There is an urgent need for increased attention to this crucial health issue from policy makers, donors, practitioners, and researchers. Policies, programmes, and research are needed to identify and address the drivers of heavy menstrual bleeding in LMICs, reduce the prevalence of heavy menstrual bleeding, and improve health outcomes. Additional population-level and multinational data are needed to estimate the prevalence of heavy menstrual bleeding at the national and global level and to provide data for decision making.

Reliable, accurate, and comparable data on heavy menstrual bleeding are needed, not only to understand the epidemiology of heavy menstrual bleeding in LMICs, but to guide policy and practice, such as that for anaemia prevention and sexual and reproductive health programmes. To address this gap, we used the SAMANTA scale, a previously validated six-item survey tool to measure heavy menstrual bleeding.^19^ We aimed to evaluate the validity of the SAMANTA scale in LMIC settings, to use this tool to calculate the prevalence of heavy menstrual bleeding among study respondents, and to examine associations between heavy menstrual bleeding and measures of wellbeing and physical health.

## Methods

### Study design

We did a cross-sectional study, using surveys implemented across two projects. The first was the Water, Sanitation, and Hygiene Partnerships and Learning for Sustainability (WASHPaLS) project, within which the Advancement of Metrics for Menstrual Hygiene Management in the Workplace study aimed to adapt or create menstrual health survey measures applicable to women working outside the home.^20^ This study included surveys done in Kathmandu, Nepal and Nairobi, Kenya between Sept 2 and Oct 9, 2021. The second was the Measuring Urban Sanitation and Empowerment (MUSE) project, which aimed to develop and validate quantitative survey instruments to measure domains and subdomains of women's empowerment in relation to sanitation in urban areas of LMICs.^21^ MUSE implemented surveys with women across eight cities in five countries (Meherpur and Saidpur, Bangladesh; Warangal, Narsapur, and Tiruchirappalli, India; Dakar, Senegal; Kampala, Uganda; and Lusaka, Zambia) between Aug 12, 2021 and June 14, 2022.

Study protocols were reviewed and approved by ethics review committees at each site: Emory University (Atlanta, GA, USA), United States International University–Africa (Nairobi, Kenya), the National Commission for Science, Technology, and Innovation (Kenya), Nepal Health Research Council (Kathmandu, Nepal), Makerere University (Kampala, Uganda), ERES Converge (Lusaka, Zambia), National Health Research Authority (Zambia), Comité National d'Ethique pour la Recherche en Santé (Dakar, Senegal), International Institute of Health Management Research (New Delhi, India), and International Training Network-Bangladesh University of Engineering and Technology (Dhaka, Bangladesh). All women provided informed consent before participation in the study.

### Participants

For both WASHPaLS and MUSE, women were included if they were aged at least 18 years and spoke the primary local language. An additional inclusion criterion in the WASHPaLS study was having experienced a menstrual period while working outside the home for cash or in-kind payments in the previous 3 months (Kenya) or 6 months (Nepal). A longer timeframe was used for Nepal due to extended COVID-19-related lockdowns, which would have prevented women from working outside the home in the months immediately before the survey. In the MUSE survey, only the subset of women who reported having a menstrual period in the previous year was asked menstruation-related questions. Among women who reported not having a menstrual period in the past year (n=1701), the most common reasons were having experienced menopause (n=507) and being pregnant or lactating (n=224).

### Procedures

The target sample size was 600 women per city for WASHPaLS and 700 women per city for MUSE. These sample sizes were calculated on the basis of standard guidelines for scale development, in which a sample size of 600 is considered sufficient.^22^ In Nepal, weather, public holidays, and pandemic-related movement restrictions created barriers to data collection, hence the target sample size was reduced to 400 women. Respondents were randomly selected within purposively selected neighbourhoods in each city, and surveys were implemented in person by trained female enumerators who were fluent in the relevant local languages. Details on survey samples and procedures are provided elsewhere^20^, ^21^ and in appendix 1 (pp 1–3).

To select a survey instrument for measurement of heavy menstrual bleeding, we conducted a literature search and identified ten candidate instruments, and the SAMANTA scale was selected as the most appropriate for our study.^19^ The SAMANTA scale consists of six questions that were developed and validated (including against clinical diagnosis of heavy menstrual bleeding) in Spain^19^ but had not been used, to our knowledge, in an LMIC setting. Further details on the selection process and criteria, instruments considered for our study, and the SAMANTA scale questions are in appendix 1 (pp 3–6).

We also added one question from the UK National Health Service (NHS) heavy periods self-assessment, ^23^ which asks whether the respondent feels excessively tired or short of breath during their menstrual period. The NHS self-assessment explains that the purpose of this question is to assess risk of anaemia caused by blood loss.^23^ Considering the well-established causal relationship between heavy menstrual bleeding and iron deficiency anaemia,^3^ this NHS screening question was added as a validation measure.

For each survey, we included modules on demographics; water, sanitation, and hygiene (WaSH) access and behaviours; and health outcomes, including one question from the Patient-Reported Outcomes Measurement Information System (PROMIS) global health subscale, which asks, “In general, how would you rate your physical health?” with response options ranging from excellent (score of 1) to poor (score of 5).^24^ The surveys also included the WHO-Five Well-Being Index (WHO-5) scale of subjective wellbeing, which uses five prompts indicating positive wellbeing (eg, “I have felt calm and relaxed”), each of which has six response options ranging from “All of the time” (score of 5) to “At no time” (score of 0).^25^

### Statistical analysis

Using the final analytic sample of all survey respondents who had answered the heavy menstrual bleeding questions, we calculated univariate descriptive statistics for all survey items that were included in the analysis.

Given that an a-priori hypothesis existed for the factor structure of the variables (based on the empirical analysis of Calaf and colleagues^19^), we used confirmatory factor analysis on the full sample to test the hypothesised one-factor structure and generate evidence on construct validity.^26^ We interpreted model fit based on the following indices and thresholds: root mean squared error of approximation (RMSEA; <0·08), comparative fit index (>0·95), Tucker–Lewis index (>0·95), and standardised root mean squared residual (SRMR; <0·08).^27^ To assess reliability, we calculated Cronbach's α as a measure of internal consistency.

We calculated scores for the SAMANTA scale and the WHO-5. For the SAMANTA scale, scoring guidelines specify that affirmative answers to two questions (experiencing menstrual bleeding for >7 days per month and being bothered by menstruation due to its abundance) each receive 3 points, while affirmative answers to all other questions each receive 1 point.^19^ These values are summed, resulting in a potential range of values for the heavy menstrual bleeding score from 0 to 10. We used the cutoff established by Calaf and colleagues,^19^ in which a score of at least 3 indicates possible heavy menstrual bleeding, to generate a binary heavy menstrual bleeding variable. For the WHO-5 scale, we summed responses for the five prompts (in which response options for each question were scored 0–5, with a potential range for the sum of 0–25) and then multiplied the sum by four (for a final score ranging from 0 to 100),^25^ whereby lower numbers indicate worse wellbeing. We calculated descriptive statistics for the prevalence of heavy menstrual bleeding and mean WHO-5 score, by city.

We assessed criterion-related validity by examining associations between the binary heavy menstrual bleeding variable and the NHS screening question, using a log-binomial regression model adjusted for neighbourhood-level clustering to estimate risk ratios (RRs). Criterion-related validity, also known as predictive or concurrent validity, is concerned with predicting an observable outcome.^22^ Heavy menstrual bleeding is a known predictor of anaemia, thus we hypothesised that the heavy menstrual bleeding measure would be positively associated with the NHS question.

We investigated associations between heavy menstrual bleeding and health outcomes. We used ordered logistic regression of self-rated physical health on heavy menstrual bleeding to estimate odds ratios (ORs) and a linear regression of the WHO-5 score on heavy menstrual bleeding to calculate β coefficients. For each regression analysis, we ran unadjusted and adjusted models. In the adjusted models, age, highest level of completed schooling (categorised as primary or less, secondary, or post-secondary), and type of menstrual material used most often (disposable pads, reusable pads, tampons, cloth, or other) were included as a-priori confounders. We theorised that each of these variables might be associated both with women's ability to manage their menstruation (and therefore their experience of heavy menstrual bleeding) and health outcomes. All models were adjusted for neighbourhood-level clustering.

We used Mplus (version 8.4) for factor analysis and Stata (version 16.1) for all other analyses.

### Role of the funding source

The funder of the MUSE project was involved in selecting study cities. Funders of the study had no other role in study design, data collection, data analysis, data interpretation, or writing of the report.

## Results

Across the ten cities, 4828 women had available data on menstruation, and the mean age of respondents was 31·5 years (SD 7·9; table 1). In all cities, with the exception of Kampala and Nairobi, most women were married. Levels of education, participation in income generating activities, and religion varied by city. The majority of women reported using disposable pads most often during menstruation, with the exception of Meherpur and Saidpur, Bangladesh, where 224 (41·0%) of 547 women and 232 (38·7%) of 599 women reported using disposable pads, respectively (table 1).

**Table 1:** Demographic characteristics and menstrual materials used most often by women who responded to the MUSE and WASHPaLS surveys, by city

		**Dakar (n=329)**	**Kampala (n=411)**	**Kathmandu (n=420)**	**Lusaka (n=436)**	**Meherpur (n=547)**	**Nairobi (n=603)**	**Narsapur (n=461)**	**Saidpur (n=599)**	**Tiruchirappalli (n=467)**	**Warangal (n=555)**	**Total (n=4828)**
Age, years												
	Mean (SD)	31·9 (8·4)	30·2 (8·1)	32·3 (7·9)	30·8 (9·1)	32·4 (7·5)	29·4 (6·0)	31·3 (7·6)	30·2 (7·8)	32·8 (7·9)	34·4 (7·9)	31·5 (7·9)
	Range	18–50	18–50	18–52	18–56	18–52	18–53	18–49	18–50	19–50	19–55	18–56
Completed schooling												
	Primary or less	141 (42·9%)	296 (72·0%)	112 (26·7%)	270 (61·9%)	326 (59·6%)	58 (9·6%)	244 (52·9%)	287 (47·9%)	230 (49·3%)	242 (43·6%)	2206 (45·7%)
	Secondary	66 (20·1%)	82 (20·0%)	108 (25·7%)	142 (32·6%)	150 (27·4%)	314 (52·1%)	82 (17·8%)	216 (36·1%)	109 (23·3%)	153 (27·6%)	1422 (29·5%)
	Post-secondary	31 (9·4%)	27 (6·6%)	196 (46·7%)	9 (2·1%)	55 (10·1%)	231 (38·3%)	62 (13·5%)	62 (10·4%)	110 (23·6%)	88 (15·9%)	871 (18·0%)
	Data missing	91 (27·7%)	6 (1·5%)	4 (1·0%)	15 (3·4%)	16 (2·9%)	0	73 (15·8%)	34 (5·7%)	18 (3·9%)	72 (13·0%)	329 (6·8%)
Participates in income generating activities		174 (52·9%)	264 (64·2%)	212 (51·0%)	197 (45·2%)	176 (32·2%)	202 (33·5%)	461 (100·0%)	136 (22·7%)	191 (40·9%)	555 (100·0%)	2568 (53·2%)
Marital status												
	Single or never married	110 (33·4%)	100 (24·3%)	156 (37·1%)	128 (29·4%)	17 (3·1%)	232 (38·5%)	74 (16·1%)	80 (13·4%)	53 (11·3%)	31 (5·6%)	981 (20·3%)
	Married	192 (58·4%)	97 (23·6%)	245 (58·3%)	248 (56·9%)	506 (92·5%)	274 (45·4%)	367 (79·6%)	510 (85·1%)	407 (87·2%)	487 (87·7%)	3333 (69·0%)
	Unmarried and living with partner	0	137 (33·3%)	0	2 (0·5%)	0	48 (8·0%)	3 (0·7%)	0	0	20 (3·6%)	210 (4·4%)
	Divorced, separated, or widowed	27 (8·2%)	77 (18·7%)	19 (4·5%)	58 (13·3%)	24 (4·4%)	49 (8·1%)	17 (3·7%)	9 (1·5%)	7 (1·5%)	17 (3·1%)	304 (6·3%)
Religion												
	Christian (Catholic)	14 (4·3%)	131 (31·9%)	39 (9·4%)	83 (19·0%)	0	178 (29·5%)	20 (4·3%)	1 (0·2%)	29 (6·2%)	17 (3·1%)	512 (10·6%)
	Christian (Protestant)	3 (0·9%)	203 (49·4%)	5 (1·2%)	341 (78·2%)	0	360 (59·7%)	45 (9·8%)	7 (1·2%)	4 (0·9%)	34 (6·1%)	1002 (20·8%)
	Muslim	312 (94·8%)	63 (15·3%)	321 (77·3%)	1 (0·2%)	486 (88·8%)	10 (1·7%)	3 (0·7%)	570 (95·2%)	63 (13·5%)	128 (23·1%)	1957 (40·6%)
	Hindu	0	0	46 (11·1%)	0	61 (11·2%)	0	392 (85·0%)	21 (3·5%)	371 (79·4%)	374 (67·4%)	1265 (26·2%)
	Other	0	14 (3·4%)	4 (1·0%)	11 (2·5%)	0	55 (9·1%)	1 (0·2%)	0	0	2 (0·4%)	87 (1·8%)
Menstrual materials used most often												
	Cloth	7 (2·1%)	25 (6·1%)	40 (9·5%)	24 (5·5%)	178 (32·5%)	2 (0·3%)	116 (25·2%)	208 (34·7%)	34 (7·3%)	108 (19·5%)	742 (15·4%)
	Reusable pads	2 (0·6%)	27 (6·6%)	14 (3·3%)	7 (1·6%)	56 (10·2%)	41 (6·8%)	14 (3·0%)	51 (8·5%)	44 (9·4%)	2 (0·4%)	258 (5·3%)
	Disposable pads	248 (75·4%)	346 (84·2%)	335 (80·0%)	347 (79·6%)	224 (41·0%)	456 (75·6%)	330 (71·6%)	232 (38·7%)	386 (82·7%)	441 (79·5%)	3345 (69·3%)
	Tampons	43 (13·1%)	0 0·0	8 (1·9%)	2 (0·5%)	0	53 (8·8%)	0	0	0	0	106 (2·2%)
	Toilet paper	4 (1·2%)	2 (0·5%)	4 (1·0%)	5 (1·1%)	78 (14·3%)	20 (3·3%)	0	79 (13·2%)	0	0	192 (4·0%)
	Cotton wool	23 (7·0%)	5 (1·2%)	3 (0·7%)	22 (5·0%)	1 (0·2%)	13 (2·2%)	0	3 (0·5%)	3 (0·6%)	0	73 (1·5%)
	None	0	0	0	0	4 (0·7%)	1 (0·2%)	0	7 (1·2%)	0	0	12 (0·3%)
	Other	2 (0·6%)	6 (1·5%)	15 (3·6%)	29 (6·7%)	6 (1·1%)	17 (2·8%)	1 (0·2%)	19 (3·2%)	0	4 (0·7%)	99 (2·1%)

Data are n (%), unless otherwise stated. MUSE=Measuring Urban Sanitation and Empowerment. WASHPaLS=Water, Sanitation, and Hygiene Partnerships and Learning for Sustainability.

Across all cities, the proportion of women who reported that they feel excessively tired or short of breath during their menstrual period was lowest in Narsapur (98 [21·3%] of 461 women) and highest in Kathmandu (233 [55·5%] of 420 women; table 2). The majority of respondents in all cities rated their physical health as good or better (table 2). The mean WHO-5 score in each city ranged from 33·6 (SD 23·1) in Kathmandu to 76·2 (25·5) in Narsapur, with a wide range of individual scores in each city (table 2); lower WHO-5 scores indicate worse wellbeing.

**Table 2:** Health and subjective wellbeing characteristics among women who responded to MUSE and WASHPaLS surveys, by city

			**Dakar (n=329)**	**Kampala (n=411)**	**Kathmandu (n=420)**	**Lusaka (n=436)**	**Meherpur (n=547)**	**Nairobi (n=603)**	**Narsapur (n=461)**	**Saidpur (n=599)**	**Tiruchirappalli (n=467)**	**Warangal (n=555)**	**Total (n=4828)**
Feel excessively tired or short of breath during menstruation			98 (29·8%)	122 (29·7%)	233 (55·5%)	125 (28·7%)	192 (35·1%)	244 (40·5%)	98 (21·3%)	155 (25·9%)	181 (38·8%)	158 (28·5%)	1606 (33·3%)
Self-rated physical health (PROMIS)													
	Excellent		11 (3·3%)	54 (13·1%)	91 (21·9%)	133 (30·5%)	14 (2·6%)	193 (32·0%)	64 (13·9%)	27 (4·5%)	110 (23·6%)	74 (13·3%)	771 (16·0%)
	Very good		90 (27·4%)	169 (41·1%)	183 (44·0%)	115 (26·4%)	101 (18·5%)	268 (44·4%)	165 (35·8%)	99 (16·5%)	99 (21·2%)	190 (34·2%)	1479 (30·7%)
	Good		196 (59·6%)	146 (35·5%)	126 (30·3%)	107 (24·5%)	246 (45·0%)	125 (20·7%)	61 (13·2%)	223 (37·2%)	224 (48·0%)	91 (16·4%)	1545 (32·0%)
	Fair		28 (8·5%)	36 (8·8%)	14 (3·4%)	64 (14·7%)	178 (32·5%)	17 (2·8%)	167 (36·2%)	244 (40·7%)	28 (6·0%)	195 (35·1%)	971 (20·1%)
	Poor		4 (1·2%)	6 (1·5%)	2 (0·5%)	17 (3·9%)	8 (1·5%)	0	4 (0·9%)	6 (1·0%)	6 (1·3%)	5 (0·9%)	58 (1·2%)
WHO-5													
	Felt cheerful												
		At no time	0	3 (0·7%)	53 (12·7%)	4 (0·9%)	4 (0·7%)	51 (8·5%)	1 (0·2%)	2 (0·3%)	80 (17·1%)	2 (0·4%)	200 (4·1%)
		Some of the time	51 (15·5%)	45 (10·9%)	181 (43·5%)	99 (22·7%)	69 (12·6%)	183 (30·3%)	45 (9·8%)	21 (3·5%)	45 (9·6%)	73 (13·2%)	812 (16·8%)
		Less than half the time	4 (1·2%)	26 (6·3%)	88 (21·2%)	25 (5·7%)	86 (15·7%)	104 (17·2%)	29 (6·3%)	19 (3·2%)	37 (7·9%)	29 (5·2%)	447 (9·3%)
		Over half the time	16 (4·9%)	29 (7·1%)	28 (6·7%)	70 (16·1%)	114 (20·8%)	60 (10·0%)	86 (18·7%)	130 (21·7%)	42 (9·0%)	91 (16·4%)	666 (13·8%)
		Most of the time	135 (41·0%)	212 (51·6%)	58 (13·9%)	192 (44·0%)	177 (32·4%)	196 (32·5%)	70 (15·2%)	306 (51·1%)	145 (31·0%)	189 (34·1%)	1680 (34·8%)
		All of the time	123 (37·4%)	96 (23·4%)	8 (1·9%)	46 (10·6%)	97 (17·7%)	9 (1·5%)	230 (49·9%)	121 (20·2%)	118 (25·3%)	171 (30·8%)	1019 (21·1%)
	Felt calm and relaxed												
		At no time	0	2 (0·5%)	47 (11·3%)	11 (2·5%)	20 (3·7%)	52 (8·6%)	0	9 (1·5%)	75 (16·1%)	4 (0·7%)	220 (4·6%)
		Some of the time	47 (14·3%)	45 (10·9%)	200 (48·1%)	111 (25·5%)	71 (13·0%)	192 (31·8%)	45 (9·8%)	68 (11·4%)	44 (9·4%)	79 (14·2%)	902 (18·7%)
		Less than half the time	4 (1·2%)	25 (6·1%)	85 (20·4%)	35 (8·0%)	86 (15·7%)	122 (20·2%)	36 (7·8%)	22 (3·7%)	36 (7·7%)	45 (8·1%)	496 (10·3%)
		Over half the time	46 (14·0%)	35 (8·5%)	24 (5·8%)	48 (11·0%)	121 (22·1%)	61 (10·1%)	96 (20·8%)	121 (20·2%)	46 (9·9%)	89 (16·0%)	687 (14·2%)
		Most of the time	156 (47·4%)	214 (52·1%)	52 (12·5%)	191 43·8%)	170 (31·1%)	171 (28·4%)	76 (16·5%)	303 (50·6%)	150 (32·1%)	198 (35·7%)	1681 (34·9%)
		All of the time	76 (23·1%)	90 (21·9%)	8 (1·9%)	40 (9·2%)	79 (14·4%)	5 (0·8%)	208 (45·1%)	76 (12·7%)	116 (24·8%)	140 (25·2%)	838 (17·4%)
	Felt active and vigorous												
		At no time	2 (0·6%)	6 (1·5%)	70 (16·8%)	3 (0·7%)	2 (0·4%)	44 (7·3%)	1 (0·2%)	15 (2·5%)	79 (16·9%)	1 (0·2%)	223 (4·6%)
		Some of the time	42 (12·8%)	41 (10·0%)	200 (48·1%)	105 (24·1%)	57 (10·4%)	193 (32·0%)	43 (9·3%)	39 (6·5%)	29 (6·2%)	63 (11·4%)	812 (16·8%)
		Less than half the time	4 (1·2%)	26 (6·3%)	70 (16·8%)	32 (7·3%)	86 (15·7%)	138 (22·9%)	38 (8·2%)	28 (4·7%)	34 (7·3%)	39 (7·0%)	495 (10·3%)
		Over half the time	23 (7·0%)	32 (7·8%)	22 (5·3%)	64 (14·7%)	129 (23·6%)	56 (9·3%)	93 (20·2%)	109 (18·2%)	39 (8·4%)	107 (19·3%)	674 (14·0%)
		Most of the time	142 (43·2%)	209 (50·9%)	46 (11·1%)	184 (42·2%)	167 (30·5%)	167 (27·7%)	71 (15·4%)	280 (46·7%)	145 (31·0%)	187 (33·7%)	1598 (33·1%)
		All of the time	116 (35·3%)	97 (23·6%)	8 (1·9%)	48 (11·0%)	106 (19·4%)	5 (0·8%)	215 (46·6%)	128 (21·4%)	141 (30·2%)	158 (28·5%)	1022 (21·2%)
	Woke up fresh and rested												
		At no time	0	4 (1·0%)	62 (14·9%)	8 (1·8%)	11 (2·0%)	60 (10·0%)	1 (0·2%)	0	71 (15·2%)	3 (0·5%)	220 (4·6%)
		Some of the time	59 (17·9%)	56 (13·6%)	185 (44·5%)	103 (23·6%)	53 (9·7%)	177 (29·4%)	44 (9·5%)	27 (4·5%)	61 (13·1%)	76 (13·7%)	841 (17·4%)
		Less than half the time	15 (4·6%)	23 (5·6%)	85 (20·4%)	26 (6·0%)	79 (14·4%)	132 (21·9%)	43 (9·3%)	30 (5·0%)	39 (8·4%)	40 (7·2%)	512 (10·6%)
		Over half the time	33 (10·0%)	27 (6·6%)	14 (3·4%)	65 (14·9%)	115 (21·0%)	46 (7·6%)	88 (19·1%)	115 (19·2%)	21 (4·5%)	109 (19·6%)	633 (13·1%)
		Most of the time	158 (48·0%)	204 (49·6%)	61 (14·7%)	190 (43·6%)	203 (37·1%)	183 (30·3%)	71 (15·4%)	315 (52·6%)	135 (28·9%)	193 (34·8%)	1713 (35·5%)
		All of the time	64 (19·5%)	97 (23·6%)	9 (2·2%)	44 (10·1%)	86 (15·7%)	5 (0·8%)	214 (46·4%)	112 (18·7%)	140 (30·0%)	134 (24·1%)	905 (18·8%)
	Daily life filled with things that interest me												
		At no time	0	6 (1·5%)	45 (10·8%)	15 (3·4%)	8 (1·5%)	33 (5·5%)	0	5 (0·8%)	73 (15·6%)	3 (0·5%)	188 (3·9%)
		Some of the time	40 (12·2%)	97 (23·6%)	162 (38·9%)	143 (32·8%)	56 (10·2%)	178 (29·5%)	46 (10·0%)	40 (6·7%)	42 (9·0%)	70 (12·6%)	874 (18·1%)
		Less than half the time	10 (3·0%)	19 (4·6%)	90 (21·6%)	59 (13·5%)	73 (13·3%)	86 (14·3%)	44 (9·5%)	46 (7·7%)	38 (8·1%)	38 (6·8%)	503 (10·4%)
		Over half the time	29 (8·8%)	43 (10·5%)	23 (5·5%)	71 (16·3%)	140 (25·6%)	66 (10·9%)	93 (20·2%)	93 (15·5%)	30 (6·4%)	106 (19·1%)	694 (14·4%)
		Most of the time	144 (43·8%)	181 (44·0%)	80 (19·2%)	120 (27·5%)	179 (32·7%)	223 (37·0%)	67 (14·5%)	316 (52·8%)	128 (27·4%)	194 (35·0%)	1632 (33·8%)
		All of the time	106 (32·2%)	65 (15·8%)	16 (3·8%)	28 (6·4%)	91 (16·6%)	17 (2·8%)	211 (45·8%)	99 (16·5%)	156 (33·4%)	144 (25·9%)	933 (19·3%)
WHO-5 score													
		Mean (SD)	74·4 (22·7)	71·3 (22·8)	33·6 (23·1)	58·8 (21·8)	64·8 (22·8)	45·9 (23·5)	76·2 (25·5)	73·1 (17·1)	62·4 (30·8)	70·8 (23·5)	63·0 (26·7)
		Range	20–100	12–100	0–100	12–100	12–100	0–100	20–100	12–100	0–100	8–100	0–100

Data are n (%), unless otherwise stated. MUSE=Measuring Urban Sanitation and Empowerment. WASHPaLS=Water, Sanitation, and Hygiene Partnerships and Learning for Sustainability. PROMIS=Patient-Reported Outcomes Measurement Information System. WHO-5=WHO-Five Well-Being Index.

The results of confirmatory factor analysis indicated a one-factor model in which all six items loaded together, with pattern coefficients ranging from 0·470 to 0·956 (appendix 1 p 7). Fit was good for the confirmatory factor analysis model (RMSEA 0·076, comparative fit index 0·986, Tucker–Lewis index 0·977, SRMR 0·053). The reliability coefficient, α, had a value of 0·745.

For criterion-related validity, strong positive associations were identified between the NHS question on feeling tired or short of breath during one's menstrual period and the binary heavy menstrual bleeding variable. In the pooled sample of all respondents, the RR was 4·12 (95% CI 3·45–4·94). For individual cities, RRs ranged from 2·03 (1·64–2·51) in Nairobi to 10·95 (6·68–17·94) in Saidpur (appendix 1 p 7). Thus, among women who were categorised as experiencing heavy menstrual bleeding, the risk of reporting that they felt tired or short of breath during a menstrual period more than doubled among respondents in Nairobi and was even higher among respondents in all other cities.

The proportion of women who were classified as experiencing heavy menstrual bleeding was lowest in Dakar (126 [38·3%] of 329 women) and Kampala (158 [38·4%] of 411 women) and highest in Kathmandu (326 [77·6%] of 420 women; table 3). In the pooled sample, 2344 (48·6%) of 4828 women were classified as experiencing heavy menstrual bleeding. Of the six items in the SAMANTA scale, women most frequently indicated that they experienced at least 3 days of heavier bleeding per month and that their menstruation bothered them due to its abundance (table 3). Women least frequently reported that they experienced bleeding for more than 7 days per month (table 3).

**Table 3:** Responses to SAMANTA scale items and prevalence of heavy menstrual bleeding, by city

	**Dakar (n=329)**	**Kampala (n=411)**	**Kathmandu (n=420)**	**Lusaka (n=436)**	**Meherpur (n=547)**	**Nairobi (n=603)**	**Narsapur (n=461)**	**Saidpur (n=599)**	**Tiruchirappalli (n=467)**	**Warangal (n=555)**	**Total (n=4828)**
Bleeding >7 days per month	69 (21·0%)	51 (12·4%)	31 (7·4%)	65 (14·9%)	51 (9·3%)	39 (6·5%)	41 (8·9%)	39 (6·5%)	46 (9·9%)	88 (15·9%)	520 (10·8%)
≥3 days of heavier bleeding	146 (44·4%)	174 (42·3%)	220 (52·4%)	147 (33·7%)	249 (45·5%)	224 (37·1%)	217 (47·1%)	283 (47·2%)	312 (66·8%)	233 (42·0%)	2205 (45·7%)
Menstruation bothers you due to its abundance	111 (33·7%)	135 (32·8%)	266 (63·3%)	196 (45·0%)	233 (42·6%)	239 (39·6%)	185 (40·1%)	268 (44·7%)	163 (34·9%)	213 (38·4%)	2009 (41·6%)
Blood spotting on clothes at night	27 (8·2%)	70 (17·0%)	231 (55·0%)	95 (21·8%)	202 (36·9%)	259 (43·0%)	118 (25·6%)	194 (32·4%)	72 (15·4%)	172 (31·0%)	1440 (29·8%)
Worried about staining furniture	17 (5·2%)	81 (19·7%)	236 (56·2%)	118 (27·1%)	193 (35·3%)	259 (43·0%)	78 (16·9%)	146 (24·4%)	69 14·8%)	111 (20·0%)	1308 (27·1%)
Avoid some activities because of the need to change menstrual materials	17 (5·2%)	69 (16·8%)	136 (32·4%)	114 (26·1%)	127 (23·2%)	240 (39·8%)	119 (25·8%)	35 (5·8%)	81 (17·3%)	126 (22·7%)	1064 (22·0%)
Heavy menstrual bleeding (SAMANTA scale score ≥3), n (%; 95% CI*)	126 (38·3%; 25·0–53·2)	158 (38·4%; 31·9–45·4)	326 (77·6%; 68·5–84·2)	225 (51·6%; 48·6–54·6)	260 (47·5%; 43·5–51·6)	295 (48·9%; 37·7–60·1)	207 (44·9%; 37·2–53·3)	287 (47·9%; 42·9–52·9)	196 (42·0%; 33·7–50·2)	264 (47·6%; 38·4–56·7)	2344 (48·6%; 44·4–52·6)

Data are n (%), unless otherwise stated.

* Adjusted for neighbourhood-level clustering.

In the full analytic sample and five cities (Lusaka, Meherpur, Narsapur, Saidpur, and Tiruchirappalli), being categorised as experiencing heavy menstrual bleeding was associated with worse self-rated physical health. We observed a positive association in adjusted models between the PROMIS subscale item and the binary heavy menstrual bleeding variable in the pooled sample (adjusted OR [aOR] 1·27, 95% CI 1·08–1·51) and all cities, with the exception of Dakar and Nairobi (table 4). The associations between heavy menstrual bleeding and self-rated physical health were statistically significant in six of the ten cities: Dakar (aOR 0·43; 0·27–0·67), Lusaka (1·80, 1·45–2·23), Meherpur (1·56, 1·16–2·09), Narsapur (1·88, 1·21–2·93), Saidpur (1·67, 1·23–2·25), and Tiruchirappalli (1·64, 1·14–2·36).

**Table 4:** Associations between self-rated physical health and heavy menstrual bleeding, in the pooled sample and by city

	**Unadjusted**			**Adjusted**		
	n	OR (95% CI)	p value	n	OR (95% CI)	p value
Total	4828	1·21 (1·09–1·34)	<0·0001	4491	1·27 (1·08–1·51)	0·005
Dakar	328	0·45 (0·31–0·67)	<0·0001	237	0·43 (0·27–0·67)	<0·0001
Kampala	411	1·19 (0·86–1·64)	0·29	405	1·23 (0·90–1·68)	0·19
Kathmandu	419	1·20 (0·63–2·28)	0·58	419	1·00 (0·59–1·69)	1·00
Lusaka	436	1·80 (1·42–2·28)	<0·0001	421	1·80 (1·45–2·23)	<0·0001
Meherpur	547	1·72 (1·28–2·32)	<0·0001	531	1·56 (1·16–2·09)	0·003
Nairobi	604	0·90 (0·56–1·47)	0·69	603	0·90 (0·54–1·51)	0·69
Narsapur	459	1·96 (1·36–2·81)	<0·0001	387	1·88 (1·21–2·93)	0·005
Saidpur	599	1·41 (1·09–1·83)	0·023	565	1·67 (1·23–2·25)	0·001
Tiruchirappalli	458	1·52 (1·00–2·31)	0·052	440	1·64 (1·14–2·36)	0·008
Warangal	554	1·58 (0·92–2·73)	0·098	483	1·70 (0·90–3·21)	0·10

Unadjusted and adjusted ordered logistic regression models controlled for clustering; adjusted models additionally controlled for age, highest level of completed schooling, and type of menstrual material used most often. The reference category was individuals who did not experience heavy menstrual bleeding. OR=odds ratio.

In the full analytic sample and in most cities, heavy menstrual bleeding was not associated with subjective wellbeing. In adjusted models, we observed a negative but non-significant association between the continuous WHO-5 index score and the binary heavy menstrual bleeding variable in the pooled data (β –3·34, 95% CI –7·04 to 0·37) and mixed associations in individual cities (table 5). The association between the continuous WHO-5 index score and the binary heavy menstrual bleeding variable was statistically significant in Warangal only (β –8·10, –15·20 to –1·01).

**Table 5:** Associations between WHO-5 index score and heavy menstrual bleeding, in pooled sample and by city

	**Unadjusted**			**Adjusted**		
	n	β coefficient (95% CI)	p value	n	β coefficient (95% CI)	p value
Total	4815	−3·95 (−8·06 to 0·17)	0·060	4491	−3·34 (−7·04 to 0·37)	0·077
Dakar	328	3·47 (−0·75 to 7·69)	0·095	237	6·91 (−1·26 to 15·08)	0·087
Kampala	411	0·13 (−4·82 to 5·08)	0·96	405	0·23 (−4·60 to 5·08)	0·92
Kathmandu	419	7·43 (−22·15 to 37·01)	0·39	419	6·04 (−13·80 to 25·89)	0·32
Lusaka	436	−4·16 (−9·91 to 1·58)	0·14	421	−4·94 (−10·98 to 1·10)	0·097
Meherpur	547	−2·18 (−6·63 to 2·27)	0·33	531	−2·12 (−6·75 to 2·50)	0·36
Nairobi	604	7·84 (0·91 to 14·77)	0·037	603	7·71 (−0·41 to 15·83)	0·057
Narsapur	459	−2·48 (−7·35 to 2·39)	0·30	387	−1·51 (−8·02 to 5·00)	0·63
Saidpur	599	−1·47 (−4·08 to 1·14)	0·26	565	−2·35 (−5·36 to 0·67)	0·12
Tiruchirappalli	458	1·54 (−3·69 to 6·77)	0·55	440	1·38 (−4·74 to 7·49)	0·64
Warangal	554	−7·66 (−14·15 to −1·18)	0·023	483	−8·10 (−15·20 to −1·01)	0·027

Unadjusted and adjusted linear regression models controlled for clustering; adjusted models additionally controlled for age, highest level of completed schooling, and type of menstrual material used most often. The reference category was individuals who did not experience heavy menstrual bleeding. WHO-5=WHO-Five Well-Being Index.

## Discussion

Our study, to our knowledge, is the first to evaluate the validity of a heavy menstrual bleeding assessment tool in LMIC settings, assess the prevalence of heavy menstrual bleeding across settings in southern Asia and sub-Saharan Africa, and examine associations with health outcomes. Results from factor analysis confirmed a unidimensional model representing heavy menstrual bleeding, providing evidence of construct validity. The prevalence of heavy menstrual bleeding across our study populations ranged from 38·3% to 77·6%, with a pooled average of 48·6%. Additionally, in all cities, strong positive associations were identified between the presence of heavy menstrual bleeding and feeling tired or short of breath during the menstrual period, demonstrating criterion-related validity. The proportion of women who reported that they feel tired or short of breath during their menstrual period ranged from 21·3% to 55·5%. This variation could be related to differences in the underlying prevalence of anaemia across our study settings. Experiencing heavy menstrual bleeding was significantly associated with worse self-rated physical health in five of ten cities (Lusaka, Meherpur, Narsapur, Saidpur, and Tiruchirappalli). We observed mixed associations between the binary heavy menstrual bleeding variable and WHO-5 score, which represents subjective wellbeing.

The reasons underlying the high prevalence of heavy menstrual bleeding in our study population and the variation in prevalence of heavy menstrual bleeding across cities remain unclear. Biological causes of heavy menstrual bleeding include structural (eg, polyps, fibroids, and malignancies) and non-structural (eg, endometrial pathology, ovulatory dysfunction, and bleeding disorders) factors.^1^, ^2^ However, it is possible that even physiologically normal menstrual blood loss might negatively affect quality of life, leading women to be categorised as experiencing heavy menstrual bleeding. For example, in many settings, poor availability of preferred menstrual materials (including insufficient quantity and quality) might result in use of suboptimal materials. Although the majority of women in our study reported using disposable pads, the quality of pads is variable, and substantial proportions of women reported using materials such as cloth. Similarly, people who menstruate might not have access to WaSH facilities and infrastructure that meet their needs and preferences, including providing sufficient water, physical space, privacy, and disposal options.^11^, ^28^ Another issue could be that, in many settings, those who menstruate receive little practical guidance on menstrual health and hygiene.^29^ Our results indicated that the highest prevalence of heavy menstrual bleeding (77·6%) was among women in Kathmandu, Nepal, where taboos related to menstruation have been well-documented^30^ and might have contributed negatively to respondents' experiences of menstruation. Considering that heavy menstrual bleeding is inherently a subjective experience,^31^ many sociocultural, environmental, and individual-level factors might contribute to the variability observed in our study.

Our results suggest a need for immediate action by donors, policy makers and practitioners across the sexual and reproductive health, menstrual health and hygiene, and WaSH sectors, among others. Heavy menstrual bleeding, by definition, impairs quality of life and overall menstrual health.^1^, ^2^, ^32^ Individuals who experience heavy menstrual bleeding have reported having their concerns dismissed by health-care providers,^7^ suggesting a need to reform health-care services. Policy makers should ensure that heavy menstrual bleeding is prioritised in sexual and reproductive health service provision and that health-care providers, including front-line health workers, receive training on assessment and management of heavy menstrual bleeding, in line with existing guidelines.^2^, ^33^ Additional policy actions could include reducing barriers to high-quality and contextually appropriate menstrual materials, for example by eliminating taxes on menstrual products or requiring their free distribution in public toilets. Practitioners can work to improve knowledge of normal and abnormal menstrual bleeding and care of the body during menstruation and to reduce taboos, including among men and boys.^12^, ^33^ Practitioners can also collaborate with government and the private sector to promote available health services and ensure that the needs of people who menstruate are met in the workplace. Practitioners should incorporate the SAMANTA scale in data collection activities to assess the prevalence of heavy menstrual bleeding for programme design, targeting, monitoring, and evaluation purposes.

Our results also suggest several directions for future research. First, additional mixed-methods research is needed to identify the issues, including biological, economic, infrastructural, or sociocultural factors underlying the observed high prevalence of heavy menstrual bleeding in each population. Second, for cases in which heavy menstrual bleeding results from an underlying biological pathology, a better understanding of the contributions of specific pathologies and any barriers to diagnosis and treatment is needed. A systematic review identified three categories of barriers to seeking consultation for abnormal uterine bleeding: low health literacy; taboos and normalisation of symptoms; and scarcity of accessible, knowledgeable, and trusted health-care providers,^33^ all of which merit further investigation in our study populations. Third, more research is needed on linkages between heavy menstrual bleeding and outcomes related to health and productivity. Our study observed a strong association between heavy menstrual bleeding and feeling tired or short of breath, which suggests a possible relationship with anaemia and aligns with a call made in 2021 for research into the relative contribution of heavy menstrual bleeding to anaemia in LMIC populations^34^ and the WHO framework for action on anaemia reduction.^35^ We also observed that heavy menstrual bleeding was associated with worse self-rated physical health in half of the cities and worse subjective wellbeing in one of the cities in our study. Physical health and wellbeing might be influenced by many factors beyond heavy menstrual bleeding, which could explain the mixed associations in our results. Linkages between heavy menstrual bleeding and health outcomes, as well as functional outcomes related to productivity and income earning, remain an important area for future investigation.

Strengths of our study include a large and diverse sample of women from ten cities in seven countries; the use of previously validated measures such as the SAMANTA, PROMIS, and WHO-5 scales; and the additional evidence generated through our study on the validity of the SAMANTA scale. Although we were unable to validate the SAMANTA scale against a clinical diagnosis of heavy menstrual bleeding, it was originally validated in a clinical setting, and the scarcity of accessible infrastructure for clinical diagnosis in LMICs is a key reason why this survey tool is needed. Our surveys targeted respondents older than 18 years living in urban areas who identify as women, meaning that adolescents and rural populations in particular were not captured, and should be specifically included in future studies. The prevalence of heavy menstrual bleeding is potentially even higher among adolescents, and availability of adequate materials might be different in rural settings.

Overall, our study provides new data on the prevalence of heavy menstrual bleeding in LMIC populations and indicates heavy menstrual bleeding is an important problem impairing health-related quality of life among a substantial proportion of women across sub-Saharan Africa and southern Asia. Our study also provides evidence on the validity and feasibility of the SAMANTA scale for widespread use in population-level surveys, to generate data on the prevalence of heavy menstrual bleeding across a range of settings that can inform policy and programmes. Continuing to improve our understanding of the scope of heavy menstrual bleeding and its potential underlying causes is crucial for developing effective strategies to reduce the experience of heavy menstrual bleeding and its consequences.

## Data sharing

Deidentified participant data and a data dictionary will be made available upon publication. Requests for data can be made via email to the corresponding author.

## Equitable partnership declaration

## Declaration of interests

S-RP reports funding from the Bill & Melinda Gates Foundation and Australian National Health and Medical Research Council; consultancy and speaker fees from Vifor Pharma; and consultancy fees for an external advisory role to WHO, all related to anaemia research. BAC reports research and consultancy funding from the Bill & Melinda Gates Foundation (via Columbia University) related to menstruation research. All other authors declare no competing interests.

## Supplementary Materials

Supplementary appendix 1

Equitable Partnership Declaration
