# Correction to *Lancet Glob Health* 2023; 11: e1775–84

**DOI:** 10.1016/S2214-109X(23)00507-7

**Published:** 2023-11-14

*Sinharoy SS, Chery L, Patrick M, et al. Prevalence of heavy menstrual bleeding and associations with physical health and wellbeing in low-income and middle-income countries: a multinational cross-sectional study.* Lancet Glob Health *2023; **11:** e1775–84*—In Table 1 of this Article, the religion data for participants in Kathmandu have been corrected. These corrections have been made as of Nov 14, 2023.

