# Supplementary material for: Prevalence of heavy menstrual bleeding and associations with physical health and wellbeing in low-income and middle-income countries: a multinational cross-sectional study
Source: Lancet Glob Health. 2023 Oct 3;11(11):e1775–84. doi: 10.1016/S2214-109X(23)00416-3 (PMC10581908; doi:10.1016/S2214-109X(23)00416-3)
Supplement: Supplementary appendix 1 [file mmc1.pdf]

THE LANCET
Global Health

Supplementary appendix 1

This appendix formed part of the original submission and has been peer reviewed. We post it as supplied by the authors.

Supplement to: Sinharoy SS, Chery L, Patrick M, et al. Prevalence of heavy menstrual bleeding and associations with physical health and wellbeing in low-income and middle-income countries: a multinational cross-sectional study. *Lancet Glob Health* 2023; published online Oct 3. https://doi.org/10.1016/S2214-109X(23)00416-3.

Supplementary Material

Methods

Study design

Selection of cities for the WASHPaLS and MUSE projects was based on the existence of prior and ongoing research activities. For the WASHPaLS project, interventions related to menstruation in the workplace had been implemented by partner organizations in Kathmandu, Nepal and Nairobi, Kenya. Therefore, these cities were targeted for additional data collection. For the MUSE project, cities were selected in coordination with the funder, Bill & Melinda Gates Foundation (BMGF), and partner organizations based on the presence of existing BMGF-funded programs and the interest of local partners.

Within each city, neighborhoods for data collection were selected based on the needs and priorities of local partners, as described below:

- In Warangal and Narsapur, collaborators from the Administrative Staff College of India (ASCI), the local partner organization, selected neighborhoods based on the presence of active gender forums and diversity of income, topographies, and vulnerability. Vulnerability was identified based on assessments of low-income areas, which mapped social and infrastructural vulnerabilities.
- In Tiruchirappalli, based on data from the Indian Institute for Human Settlements (IIHS), the Emory team selected a mix of slum and non-slum neighborhoods with varied access to community toilets and seats designated for the use of women or friendly to those with disabilities.
- In Lusaka, collaborators from the Lusaka Water Supply and Sanitation Company (LWSC) selected townships of different sizes which had often been left out of past data collection efforts.

- For each of Kampala’s five districts, a BMGF-funded Community-Wide Inclusive Sanitation model parish and a non-model parish of comparable socio-economic levels, matched based on slum or non-slum status, were selected.
- In Dakar, based on data and recommendations from collaborators at the Office National de L’Assainissement du Sénégal / National Sanitation Office of Senegal (ONAS) and Speak Up Africa, the Emory team selected neighborhoods such that 30% of neighborhoods had received sanitation infrastructure projects in the past, and 70% were receiving new sanitation interventions.
- In Meherpur, neighborhood selection was determined by a multi-stage cluster sampling procedure where first, 34 *mohallas* (administrative blocks), drawn using standard probability proportional to size method, were considered as the primary sampling unit and divided into clusters of about 150 households and second, one cluster from each Mohalla was selected using simple random sampling, with households from that cluster designated as the sampling unit.
- In Saidpur, there was no neighborhood selection; data collection occurred in all *mohallas*.
- In Kathmandu, districts were selected with consultation from country experts based on variability of occupation, sectors of work, and socio-economic status; two districts have greater concentration of service-oriented workplaces, traders, farmers, and informal workplaces such as markets, and the third district has more factories and industrial work producing items such as ready-made garments, cement, bricks, plastics, and polymers.
- In Nairobi, four sub-counties were selected that each have variability in job types, sectors of work, and socioeconomic status, though three of sub-counties have greater access to industrialized workplaces and offices.

Participants and procedures

The process for participant selection was the same in all ten cities and involved random door-knocking on every third door. In areas where individuals were likely to live in compounds/plots, such as Lusaka, the plot was treated as a household, and data collectors tried to recruit from one home within the plot. Female data collectors who had been trained on the survey instrument and who were fluent in the local languages (Nepali in Kathmandu, Luganda in Kampala, Swahili in Nairobi, Tamil in Tiruchirappalli, Telegu in Narsapur and Warangal, Nyanja in Lusaka, Bengali in Bangladesh, and Wolof and French in Dakar) administered the survey with one adult woman participant per household, using tablets programmed with the ONA survey platform.

Data collection instruments

To select an instrument for inclusion in our study, we began with a literature search and identified existing instruments (Supplemental Table 1). We eliminated from consideration any approaches that relied on laboratory-based measurement of blood volume from menstrual products, given impracticality for assessment at scale in multiple settings. We also eliminated instruments that we deemed to be overly long and burdensome for large-scale survey implementation (i.e., those that included 20 or more survey prompts); instruments that asked about disposable pads and tampons, which may be unavailable or not widely used in some LMIC settings; and those that were pictorial as they have been determined to be unsuitable for widespread use (1).

Supplemental Table 1. Sources for survey instruments considered, and determination about appropriateness for implementation, in our study

**Citation**	**Determination**
Shaw RW, Brickley MR, Evans L, Edwards MJ. Perceptions of women on the impact of menorrhagia on their health using multi-attribute utility assessment. BJOG: An International Journal of Obstetrics & Gynaecology. 1998 Nov;105(11):1155-9.	Questionnaire designed for women who were already identified as needing treatment for menorrhagia; focused on outcomes rather than assessment of HMB
Bushnell DM, Martin ML, Moore KA, Richter HE, Rubin A, Patrick DL. Menorrhagia Impact Questionnaire: assessing the influence of heavy menstrual bleeding on quality of life. Current medical research and opinion. 2010 Dec 1;26(12):2745-55.	Questionnaire designed for women who were already identified as needing treatment for menorrhagia; focused on outcomes rather than assessment of HMB
Calaf J, Cancelo MJ, Andeyro M, Jiménez JM, Perelló J, Correa M, Parera N, Lete LI, Calvo A, Doval JL, Duarte R. Development and psychometric validation of a screening questionnaire to detect excessive menstrual blood loss that interferes in quality of life: the SAMANTA questionnaire. Journal of Women's Health. 2020 Oct 1;29(10):1292-302.	Selected for inclusion in study
Su S, Yang X, Su Q, Zhao Y. Prevalence and knowledge of heavy menstrual bleeding among gynecology outpatients by scanning a WeChat QR Code. PloS one. 2020 Apr 2;15(4):e0229123.	Questionnaire requires use of a pictorial method (the Pictorial Blood Loss Assessment Chart)
Matteson KA, Scott DM, Raker CA, Clark MA. The menstrual bleeding questionnaire: development and validation of a comprehensive patient-reported outcome instrument for heavy menstrual bleeding. BJOG: An International Journal of Obstetrics & Gynaecology. 2015 Apr;122(5):681-9.	Questionnaire includes ≥20 questions
Toxqui L, Pérez-Granados AM, Blanco-Rojo R, Wright I, Vaquero MP. A simple and feasible questionnaire to estimate menstrual blood loss: relationship with hematological and gynecological parameters in young women. BMC women's health. 2014 Dec;14(1):1-6.	Questionnaire designed to assess menstrual blood loss, not heavy menstrual bleeding, and relies on count of pads and tampons used
Ruta DA, Garratt AM, Chadha YC, Flett GM, Hall MH, Russell IT. Assessment of patients with menorrhagia: how valid is a structured clinical history as a measure of health status?. Quality of Life Research. 1995 Feb;4(1):33-40.	Questionnaire asks about number of pads and tampons used, also about interference with sex life, which may be culturally sensitive
Lamping DL, Rowe P, Clarke A, Black N, Lessof L. Development and validation of the menorrhagia outcomes questionnaire. BJOG: An International Journal of Obstetrics & Gynaecology. 1998 Jul;105(7):766-79.	Questionnaire designed for women who were already identified as needing treatment for menorrhagia; focused on outcomes rather than assessment of HMB
Philipp CS, Faiz A, Heit JA, Kouides PA, Lukes A, Stein SF, Byams V, Miller CH, Kulkarni R. Evaluation of a screening tool for bleeding disorders in a US multisite cohort of women with menorrhagia. American journal of obstetrics and gynecology. 2011 Mar 1;204(3):209-e1.	Questionnaire designed for women who were already identified as needing treatment for menorrhagia, to screen for underlying bleeding disorders
UK National Health Service. Heavy periods self-assessment 2021 [updated 03 November 2021; cited 2022 09 September]. Available from: https://www.nhs.uk/conditions/heavy-periods/.	Questionnaire includes specific references to pads, tampons, and menstrual cups; selected one question for inclusion as a validation measure

Based on our review, we selected the SAMANTA scale (2), which includes six questions, all with binary (yes/no) response options, as shown in Supplementary Table 2.

Supplemental Table 2. SAMANTA scale items

Item number	Question
1	Do you experience menstrual bleeding during more than 7 days per month?
2	Do you experience 3 or more days of heavier menstrual bleeding during your menstrual period?
3	In general, does menstruation bother you due to its abundance?
4	During any of these heavier menstrual bleeding days do you spot your clothes at night; or would you spot them if you did not use double protection/did not change your clothes during the night?
5	During these heavier menstrual bleeding days, are you worried about staining the chair, sofa, etc.?
6	In general, during these heavier menstrual bleeding days, do you aovid, as far as possible, some activities, trips, or leisure-time plans because you frequently need to change your tampon or sanitary towel?

Surveys were translated into the local language(s) of each city. The translated survey instruments were then carefully reviewed during training with data collectors, who were encouraged to provide feedback and suggest changes to language. Adjustments to the translations were made accordingly, before finalizing the instrument for use in the field.

We pilot tested the surveys in all cities and additionally conducted cognitive interviews using the SAMANTA scale items with 15 women in one city, Dakar. Cognitive interviewing is a qualitative approach used to identify discrepancies between how researchers ask questions and how respondents interpret and understand them (3, 4). Cognitive interviews involve administering survey instruments and asking open-ended questions about the survey questions and responses, to identify how the participants understand the questions and why they provide their selected responses. The process can illuminate situations in which respondents may have difficulty understanding the questions or selecting an appropriate response option. It can also help researchers to identify survey questions that may be culturally sensitive or not relevant in a given context. Finally, the data from cognitive interviews can inform survey revisions, including eliminating questions, rewording questions to be clearer, adding context-specific examples to clarify questions, and amending response options. The cognitive interviews in Dakar were conducted by trained enumerators who asked consenting participants each survey question, provided response options, and then asked participants to think aloud about their response. Enumerators could probe to assess whether participants understood and interpreted questions as

intended. Respondents’ answers and enumerators’ impressions and observations were recorded in field notes. No revisions to the survey questions were deemed necessary, based on the pilot tests or cognitive interviews.

Supplementary Table 3. Standardized pattern coefficients from confirmatory factor analysis of SAMANTA scale

Item number	Standardized pattern coefficients
1	0.470
2	0.587
3	0.674
4	0.924
5	0.956
6	0.749

Supplementary Table 4. Parameter estimates from log-binomial regression models of feeling excessively tired or short of breath during menstrual period on HMB, in pooled sample and by city (Reference: not experiencing HMB)

Pooled	N	Risk ratio (95% CI)	p-value
	4815	4.12 (3.45, 4.94)	<0.001
**By City**			
Dakar	328	3.20 (1.54, 6.64)	0.002
Kampala	411	4.91 (3.63, 6.65)	<0.001
Kathmandu	419	2.54 (1.98, 3.26)	<0.001
Lusaka	436	3.57 (2.59, 4.93)	<0.001
Meherpur	547	6.75 (4.75, 9.89)	<0.001
Nairobi	604	2.03 (1.64, 2.51)	<0.001
Narsapur	459	8.72 (5.27, 14.43)	<0.001
Saidpur	599	10.95 (6.68, 17.94)	<0.001
Tiruchirappalli	458	3.57 (2.72, 4.71)	<0.001
Warangal	554	4.92 (2.72, 8.90)	<0.001
Note: Models adjusted for clustering			

References

1. Magnay JL, O'Brien S, Gerlinger C, Seitz C. Pictorial methods to assess heavy menstrual bleeding in research and clinical practice: a systematic literature review. BMC Women's Health. 2020;20(1):24.
2. Calaf J, Cancelo MJ, Andeyro M, Jiménez JM, Perelló J, Correa M, et al. Development and psychometric validation of a screening questionnaire to detect excessive menstrual blood loss that interferes in quality of life: the SAMANTA questionnaire. Journal of Women's Health. 2020;29(10):1292-302.
3. Willis GB, Miller K. Cross-cultural cognitive interviewing: Seeking comparability and enhancing understanding. Field methods. 2011;23(4):331-41.
4. DeVellis RF. Scale Development: Theory and Applications. 4th ed. Thousand Oaks, CA: SAGE; 2017. 262 p.
