# Supplementary material for: Prevalence of heavy menstrual bleeding and associations with physical health and wellbeing in low-income and middle-income countries: a multinational cross-sectional study
Source: Lancet Glob Health. 2023 Oct 3;11(11):e1775–84. doi: 10.1016/S2214-109X(23)00416-3 (PMC10581908; doi:10.1016/S2214-109X(23)00416-3)
Supplement: Equitable Partnership Declaration [file mmc2.pdf]

THE LANCET
Global Health

Supplementary appendix 2

This Equitable Partnership Declaration (EPD) was submitted by the authors, and we reproduce it as supplied. It has not been peer reviewed. *The Lancet*'s editorial processes have not been applied to the EPD.

Supplement to: Sinharoy SS, Chery L, Patrick M, et al. Prevalence of heavy menstrual bleeding and associations with physical health and wellbeing in low-income and middle-income countries: a multinational cross-sectional study. *Lancet Glob Health* 2023; published online Oct 3. https://doi.org/10.1016/S2214-109X(23)00416-3.

Equitable Partnership Declaration questions

Researcher considerations

1. Please detail the involvement that researchers who are based in the region(s) of study had during a) study design; b) clinical study processes, such as processing blood samples, prescribing medication, or patient recruitment; c) data interpretation; and d) manuscript preparation, commenting on all aspects. If they were not involved in any of these aspects, please explain why.

   This question is intended for international partnerships; if all your authors are based in the area of study, this question is not applicable.

   This should include a thorough description of their leadership role(s) in the study. Are local researchers named in the author list or the acknowledgements, or are they not mentioned at all (and, if not, why)? Please also describe the involvement of early career researchers based in the location of the study. Some of this information might be repeated from the Contributors section in the manuscript. Note: we adhere to ICMJE authorship criteria when deciding who should be named on a paper.

**a) Study design**<br><br>This research relies primarily on a scale deployed as part of two different projects, referred to throughout as MUSE and WASHPaLS.<br><br>For MUSE, researchers and other relevant stakeholders (including from local government agencies) in the regions of study reviewed the survey instruments and were involved in the selection of target neighbourhoods for data collection. In some cases, these local partners selected the target neighbourhoods themselves; in others, they provided input and guidance as part of the selection process. Among co-authors on the current manuscript, Jenala Chipungu and Beatrice Chiwala-Chibwe are based in Zambia; Niladri Chakraborti and Malini Reddy are based in India; and Tanvir Ahmed is based in Bangladesh. They all reviewed the MUSE survey instrument, suggested edits, identified neighbourhoods or advised on neighbourhood selection, and provided input on participant sampling. In addition, Prof. Ahmed was the local Principal Investigator (PI) for the MUSE project in Bangladesh and received a grant directly from the Bill & Melinda Gates Foundation to cover the costs of data collection for MUSE. As such, he had a leadership role that included full oversight of the study activities in Bangladesh.<br><br>For WASHPaLS, researchers in the regions of study were involved from the beginning of the study design process. Among co-authors on the current manuscript, Aparna Stephen and Anupama Ramaswamy are based in India. They co-authored the grant proposal to secure funding for this work and were equal partners in developing the study instrument and designing the study protocol, including data collection type and sampling strategy. They also led engagement with several key stakeholders in each study location to discuss the study aims and relevance to country priorities and initiatives. In their leadership capacity, they had total control over local funding allocation.<br><br>Among the authors listed above, Ms. Chipungu and Ms. Stephen are early career researchers.
**b) Clinical study processes:** Our study did not involve clinical processes. Our study involved cross-sectional surveys. Ms. Chipungu, Ms. Ramaswamy, and Ms. Stephen were involved in data collection through training and management of enumeration teams during data collection. Co-

authors also facilitated interactions and permission from city governments to conduct the study. Prof. Ahmed, as site PI in Bangladesh, had a leadership role in data collection, including coordinating the partner organizations that carried out data collection activities in Bangladesh.
**c) Data interpretation:** All authors were involved in data interpretation.
**d) Manuscript preparation:** All authors were involved in manuscript preparation, specifically in the review and editing of the draft manuscript.

2. Were the data used in your study collected by authors named on the paper, or have they been extracted from a source such as a national survey? ie, is this a secondary analysis of data that were not collected by the authors of this paper. If the authors of this paper were not involved in data collection, how were data interpreted with sufficient contextual knowledge?

   The Lancet Global Health *believe contextual understanding is crucial for informed data analysis and interpretation.*

Our study involved primary data collection. The data were collected by enumerators who were hired and trained for the purpose of this study. As described above, some authors of this paper, namely Ms. Chipungu, Ms. Ramaswamy, and Ms. Stephen, were involved in training and supported the management of data collection teams. In addition, Mr. Chakraborty, Dr. Reddy, Prof. Ahmed, and Ms. Chiwala had access to the results, are familiar with the local contexts, and provided contextual understanding of the neighbourhoods and cities where data was collected for data interpretation.

3. How was funding used to remunerate and enhance the skills of researchers and institutions based in the area(s) of study? And how was funding used to improve research infrastructure in the area of study?

   Potentially effective investments into long-term skills and opportunities within institutions could include training or mentorship in analytical techniques and manuscript writing, opportunities to lead all or specific aspects of the study, financial remuneration rather than requiring volunteers, and other professional development and educational opportunities.

   Improvements to research infrastructure could be funding of extended trial designs (such as platform trials) and use of master protocols to enable these designs, establishment of long-term contracts for research staff, building research facilities, and local control of funding allocation.

Remuneration: Authors (researchers) and data collectors were paid for their effort on the study through grants from the Bill & Melinda Gates Foundation (MUSE) and USAID (WASHPaLS). Payment took different forms, including through short-term contracts, consultancies, and permanent salaried positions, depending on the capacity in which individuals were involved in the projects.

Skill development: Funding was used to hire and train local data collection teams in each city. Training topics included research ethics, privacy, confidentiality, subject matter information, and survey administration. Earlier phases in the work also included training in cognitive interviews, which local partners were invited to join, so that they could learn a data collection method that is not as widespread. Local partners were engaged in manuscript development and invited to join in conference presentations and manuscript writing.
Research infrastructure: Our research comprised cross-sectional surveys, and each city's data collection lasted approximately 6-8 weeks. Therefore, investments such as establishing long-term contracts, building infrastructure, and funding extended trials were not feasible within our project period.

4. How did you safeguard the researchers who implemented the study?

Please describe how you guaranteed safe working conditions for study staff, including provision of appropriate personal protective equipment, protection from violence, and prevention of overworking.

To safeguard both data collectors and study participants from COVID-19, all participants were asked screening questions related to COVID-19 symptoms before data collection began, and the survey was ended if participants reported that they were experiencing any of the listed symptoms. In addition, data collectors were provided and required to wear masks, and they were also provided hand sanitizer. Training included information on protection from COVID-19.<br><br>To protect data collectors from violence, we advised study staff and managers that data collectors' safety in the field was a priority and that they should immediately leave any environment where they felt unsafe, whether that was a house in the middle of data collection or a community. In two cities, we asked local partners to choose a new neighbourhood for data collection because the team reported feeling unsafe. We avoided data collection after dark, and data collectors worked in pairs so that they would always be near another team member. Finally, local community leaders (including local elected representatives) were informed of the planned data collection activities so that they could facilitate the smooth and safe implementation of the surveys.<br><br>To prevent overwork, we reduced the planned number of surveys asked in a day to ensure reasonable working hours.

<u>Benefits to the communities and regions of study</u>

5. How does the study address the research and policy priorities of its location?

How were the local priorities determined and then used to inform the research question? Who decided which priorities to take forward? Which elements of the study address those priorities?

For the MUSE project, we worked with representatives of local and national government agencies in all five countries where data collection took place. We began consulting with these government stakeholders during the study design phase, to align the research with local policy priorities. Specifically, the following local government agencies were involved: Tiruchirappalli City

Corporation (TCC); Lusaka Water Supply and Sanitation Company (LWSC); Kampala Capital City Authority (KCCA); Office National de l'Assainissement du Sénégal / National Office of Sanitation of Senegal (ONAS); and the Department of Public Health Engineering (DPHE) in Bangladesh. All were provided with copies of the survey instrument and asked to provide feedback and to propose additional questions that would allow them to gather data related to their priorities. As a result of these consultations, we included questions in the surveys in some cities that were newly developed based on requests from local stakeholders, such as questions about sanitation infrastructure that were not directly relevant to our study objective. Local partners also selected or advised on the selection of neighbourhoods that were of interest for their own programs. In some cities, that meant choosing some neighbourhoods that had received government programs and some that had not yet received those programs, or choosing to collect data among neighbourhoods with specific infrastructural or cultural characteristics.

For WASHPaLS, we held local high-level consultations with key stakeholders in each country, to ensure relevance of the data at the national level. We consulted with stakeholders from government agencies and non-governmental organizations to understand local policy and measurement priorities and data collection activities related to menstrual health and hygiene in the workplace.

6. How will research products be shared in the community of study?

For instance, will you be providing written or oral layperson summaries for non-academic information sharing? Will study data be made available to institutions in the region(s) of study? The Lancet Global Health *encourages authors to translate the summary (abstract) into relevant languages after paper editing; do you intend to translate your summary?*

Results from the two projects have been shared broadly with partners through briefing papers, presentations, and tables of relevant statistics. We also plan to produce a one-page summary of this paper for sharing with non-academic audiences.

For MUSE, we have produced customized four- to seven-page briefing papers for each of the eight cities in which data collection took place and have shared these with local partners, some of whom have organized local events to further disseminate results. Each of the briefing papers presents data from that city, with descriptions written in layperson terms and accompanied by figures (e.g. bar graphs, pie charts) that are designed to be easily read by a layperson. The briefing paper for Dakar has additionally been translated into French for local stakeholders. For three cities where MUSE partners have expressed an interest, we have also paid a graphic designer to distil relevant statistics into one-page infographics to be shared with relevant local stakeholders. We have worked with partners in Uganda, Zambia, and India who wished to share results with other stakeholders by drafting slides for presentations and creating content when requested. Finally, additional information (including other reports and publications from this project) are publicly available to anyone globally on the MUSE website at https://www.museproject.org/.

For WASHPaLS, we shared results through presentations and a 184-page report that is publicly available at
https://www.globalwaters.org/sites/default/files/washpals_mhh_metrics_report_final_jan2022_1.27_final_1.pdf.

MUSE data has been made available to all partner institutions. The data will also be published to Emory Dataverse, where it will be accessible to anyone, include institutions in the regions of study. In addition, for partners in two cities who requested that the data be shared with them in the form of tables, we have calculated descriptive statistics for all variables in the MUSE survey and shared these with the partners.

WASHPaLS was funded by USAID, and in line with the funder's requirements, the data have been submitted for publication at the USAID Development Data Library (DDL).

We intend to translate our summary (abstract) into relevant languages.

7. How were individuals, communities, and environments protected from harm?

a) *How did you ensure that sensitive patient data was handled safely and respectfully? Was there any potential for stigma or discrimination against participants arising from any of the procedures or outcomes of the study?*

No patient data was handled. Participant interactions were in the form of surveys. The data presented in the current manuscript were collected from women. Given the target population, we only hired women as data collectors, to minimize the risk of potential harm or social repercussions introduced through participants having study interactions or being alone with men. Data collection teams were trained on protecting privacy and confidentiality of participants. Approval was obtained from relevant local ethics committees, and the research was conducted in line with standard ethical principles.

b) *Might any of the tests be experienced as invasive or culturally insensitive?*

We did not conduct any tests. We conducted surveys, which were reviewed by local ethics boards, local partners, and data collection teams prior to survey administration. None of the survey questions were invasive or culturally insensitive.

c) *How did you determine that work was sensitive to traditions, restrictions, and considerations of all cultural and religious groups in the study population?*

Data collection schedules accommodated local holidays and festivals. Neighbourhoods were selected for diversity, including diversity of religion, ethnicities, and cultural groups. Questions that were seen as potentially stigmatizing or sensitive by local partners were dropped or adapted. Local ethical review boards reviewed the study in each country.

d) *Were biowaste and radioactive waste disposed of in accordance with local laws?*

N/A

e) *Were any structures built that would have impacted members of the community or the environment (such as handwashing facilities in a public space)? If so, how did you ensure that you had appropriate community buy-in?*

N/A

f) *How might the study have impacted existing health-care resources (such as staff workloads, use of equipment that is typically employed elsewhere, or reallocation of public funds)?*

N/A

8. Finally, please provide the title (eg, Dr/Prof, Mr/Mrs/Ms/Mx), name, and email address of an author who can be contacted about this statement. This can be the corresponding author.

Name: Dr. Sheela Sinharoy
Email: sheela.sinharoy@emory.edu
